# Expert consensus on intentional tooth replantation

**DOI:** 10.1038/s41368-024-00337-5

**Published:** 2025-03-03

**Authors:** Zhengmei Lin, Dingming Huang, Shuheng Huang, Zhi Chen, Qing Yu, Benxiang Hou, Lihong Qiu, Wenxia Chen, Jiyao Li, Xiaoyan Wang, Zhengwei Huang, Jinhua Yu, Jin Zhao, Yihuai Pan, Shuang Pan, Deqin Yang, Weidong Niu, Qi Zhang, Shuli Deng, Jingzhi Ma, Xiuping Meng, Jian Yang, Jiayuan Wu, Lan Zhang, Jin Zhang, Xiaoli Xie, Jinpu Chu, Kehua Que, Xuejun Ge, Xiaojing Huang, Zhe Ma, Lin Yue, Xuedong Zhou, Junqi Ling

**Affiliations:** 1 Department of Operative Dentistry and Endodontics, Hospital of Stomatology, Guanghua School of Stomatology, Sun Yat-Sen University & Guangdong Provincial Key Laboratory of Stomatology, Guangzhou, China; 2 State Key Laboratory of Oral Diseases & National Clinical Research Center for Oral Diseases & Department of Cariology and Endodontics, West China Hospital of Stomatology, Sichuan University, Chengdu, China; 3 State Key Laboratory of Oral & Maxillofacial Reconstruction and Regeneration, Key Laboratory of Oral Biomedicine Ministry of Education, Hubei Key Laboratory of Stomatology, School & Hospital of Stomatology, Wuhan University, Wuhan, China; 4 State Key Laboratory of Oral & Maxillofacial Reconstruction and Regeneration, National Clinical Research Center for Oral Diseases, Shanxi Key Laboratory of Oral Diseases, Department of Operative Dentistry & Endodontics, School of Stomatology, The Fourth Military Medical University, Xi’an, China; 5 Center for Microscope Enhanced Dentistry, Beijing Stomatological Hospital, Capital Medical University, Beijing, China; 6 Department of Endodontics, School of Stomatology, China Medical University, Shenyang, China; 7 College & Hospital of Stomatology, Guangxi Medical University, Nanning, Guangxi China; 8 Department of Cariology and Endodontology, Peking University School and Hospital of Stomatology & National Clinical Research Center for Oral Diseases & National Engineering Research of Oral Biomaterials and Digital Medical Devices & Beijing Key Laboratory of Digital Stomatology, Peking University, Beijing, China; 9 Department of Endodontics, Shanghai Ninth People’s Hospital, Shanghai Jiao Tong University School of Medicine, College of Stomatology, Shanghai Jiao Tong University, National Clinical Research Center for Oral Diseases, National Center for Stomatology, Shanghai Key Laboratory of Stomatology, Shanghai, China; 10 Department of Endodontics, Jiangsu Key Laboratory of Oral Diseases, Affiliated Hospital of Stomatology, Nanjing Medical University, Nanjing, China; 11 Department of Endodontics, The First Affiliated Hospital of Xinjiang Medical University, Urumqi, China; 12 Department of Endodontics, School and Hospital of Stomatology, Wenzhou Medical University, Wenzhou, China; 13 Department of Endodontics, The First Affiliated Hospital of Harbin Medical University & Department of Endodontics, School of Stomatology, Harbin Medical University, Harbin, China; 14 Department of Conservative Dentistry and Endodontics, Shanghai Stomatological Hospital & School of Stomatology, Shanghai Key Laboratory of Craniomaxillofacial Development and Diseases, Fudan University, Shanghai, China; 15 School of Stomatology, Dalian Medical University, Dalian, Liaoning China; 16 Department of Endodontics, Stomatological Hospital and Dental School of Tongji University, Shanghai Engineering Research Center of Tooth Restoration and Regeneration, Shanghai, China; 17 Stomatology Hospital, School of Stomatology, Zhejiang University School of Medicine, Zhejiang Provincial Clinical Research Center for Oral Diseases, Key Laboratory of Oral Biomedical Research of Zhejiang Province, Cancer Center of Zhejiang University, Hangzhou, China; 18 Department of Stomatology, Union Hospital, Tongji Medical College, Huazhong University of Science and Technology, Wuhan, China; 19 Hospital of Stomatology, Jilin University, Changchun, China; 20 Department of Endodontics, The Affiliated Stomatological Hospital of Nanchang University, Nanchang, China; 21 Key Laboratory of Oral Disease Research, School of Stomatology, Zunyi Medical University, Zunyi, China; 22 Shandong University, School of Stomatology, Jinan, Shandong China; 23 Department of Endodontology, Hunan Xiangya Stomatological Hospital, Central South University, Changsha, China; 24 The First Affiliated Hospital of Zhengzhou University, Zhengzhou, China; 25 Department of Endodontics, College of Stomatology, Tianjin Medical University, Tianjin, China; 26 Shanxi Province Key Laboratory of Oral Diseases Prevention and New Materials, Shanxi Medical University School and Hospital of Stomatology, Taiyuan, Shanxi China; 27 Fujian Key Laboratory of Oral Disease & Fujian Provincial Engineering Research Center of Oral Biomaterial & Stomatological Key Lab of Fujian College and University, School and Hospital of Stomatology, Fujian Medical University, Fuzhou, China; 28 Department of Preventive Dentistry, Hebei Key Laboratory of Stomatology, Hebei Clinical Research Center for Oral Diseases, School and Hospital of Stomatology, Hebei Medical University, Shijiazhuang, Hebei, China

**Keywords:** Endodontics, Oral surgery

## Abstract

Intentional tooth replantation (ITR) is an advanced treatment modality and the procedure of last resort for preserving teeth with inaccessible endodontic or resorptive lesions. ITR is defined as the deliberate extraction of a tooth; evaluation of the root surface, endodontic manipulation, and repair; and placement of the tooth back into its original socket. Case reports, case series, cohort studies, and randomized controlled trials have demonstrated the efficacy of ITR in the retention of natural teeth that are untreatable or difficult to manage with root canal treatment or endodontic microsurgery. However, variations in clinical protocols for ITR exist due to the empirical nature of the original protocols and rapid advancements in the field of oral biology and dental materials. This heterogeneity in protocols may cause confusion among dental practitioners; therefore, guidelines and considerations for ITR should be explicated. This expert consensus discusses the biological foundation of ITR, the available clinical protocols and current status of ITR in treating teeth with refractory apical periodontitis or anatomical aberration, and the main complications of this treatment, aiming to refine the clinical management of ITR in accordance with the progress of basic research and clinical studies; the findings suggest that ITR may become a more consistent evidence-based option in dental treatment.

## Introduction

Pupal and periradicular pathosis, caused by caries, trauma, or dental abnormalities, is a common disease requiring dental treatment in the clinic. Root canal treatment (RCT) is the conventional option for managing endodontic diseases, with a pooled success rate of 92.6%.^1,2^ For teeth with persistent pain or periapical lesions after RCT, nonsurgical retreatment and endodontic microsurgery (EMS) should always be considered. However, in specific cases with inaccessible endodontic or resorptive lesions or lesions adjacent to vital structures, endodontic retreatment and EMS are impractical. In these situations, intentional tooth replantation (ITR) may be the last resort for preserving natural teeth.^3^

ITR is one of the oldest known methods for the treatment of endodontic diseases, dating as far back as the 10th century, when the Chinese doctor Huaiyin Wang described it in his medical classic “*General Records of Holy Universal Relief*”. Specifically, he stated that avulsed teeth should be replanted into the alveolar socket and fixed with wrought copper for approximately five days for stabilization. Subsequently, Abulcasis, a famous Arab surgeon, performed a classic ITR procedure in the 11th century, and since then, multiple accounts of ITR have been reported. ITR was formally defined as “the purposeful removal of a tooth and its almost immediate replacement, with the objective of obturating the canals apically when the tooth is out of the alveolar socket” by Grossman in 1982.^4^ The evolution of this procedure has involved the modification of techniques for tooth extraction, root-end resection and preparation; the handling of the tooth during surgical manipulation; and the materials used for root-end filling. Notably, with the development of stem cell therapy, microtechnology and osteo-inductive biomaterials, the overall success rate of ITR has significantly increased to 89.1% according to rigorous evidence-based analysis.^5^ Choi reported that the success rates of ITR at 4 years post-operation were 91.2%,^6^ while Pisano systematically evaluated the overall success rate of ITR in single and multiple teeth as 86.7%.^7^ The efficacy of ITR in the retention of refractory and untreatable teeth that cannot be managed with conventional methods has gradually been accepted by more clinicians.^8,9^

To date, more than 300 articles have been published, as found in PubMed through a search with the keywords “ITR”, “tooth replantation”, “tooth transplantation” and “autotransplantation of tooth”. The article types included basic studies, case reports, case series, retrospective studies, prospective clinical trials, reviews and guidelines; the most common article types were case reports and reviews. At present, two guidelines on ITR are available for dental practitioners. The American Association of Endodontists (AAE) published clinical guidelines for ITR in 2019, briefly outlining the indications, clinical procedures, and treatment goals of ITR. The European Society of Endodontology (ESE) position statement was published in 2021 and provides additional procedure details, including considerations in case selection, clinical protocols, and follow-up requirements.^10^ Chinese scholars have conducted extensive exploration of ITR in both preclinical and clinical studies, gradually guiding primary care physicians to implement this technique.^11–15^ This expert consensus systematically described the biological foundation, case selection, clinical protocol and outcome management of ITR, aiming to standardize the clinical practice of this technique and extend tooth longevity.

## Biological foundation of ITR

### Etiology

Bacteria within the root canal are the etiological basis for periapical tissue lesions. When the affected tooth exhibits abnormal dental structures or complex root canal systems, conventional endodontic treatment often fails to eradicate infection inside and outside the root canal, leading to treatment failure.^16^ The advantages of ITR include the ability to thoroughly inspect and treat the root surface and periradicular area under direct vision and the ability to address areas that are difficult to manage with conventional root canal treatment or EMS, thus eliminating various etiological factors, controlling infection, and preserving the affected tooth.^17^ Advancements in modern EMS have provided substantial technical possibilities for enhancing the efficacy of ITR.^18^

### Tissue preservation and regeneration

The proper preservation and regeneration of periodontal tissues are critical for healing after ITR.^19^ The periodontal ligament is a dense connective tissue located between the tooth root and alveolar bone, with one end embedded in the cementum and the other extending into the alveolar bone, anchoring the tooth root and alleviating chewing pressure. The periodontal ligament is mainly composed of collagen fibers, matrix, periodontal ligament cells, cementoblasts, and neurovascular components. When a tooth is extracted, its periodontal ligament is usually bisected, with the inner layer attaching to the root surface and the rest remaining within the alveolar socket. Healing of the traumatized periodontal ligament involves two processes: revascularization of injured tissues and formation of new tissue. Initially, after extraction, macrophages, endothelial cells, fibroblasts, and other cells migrate in a coordinated manner to the injured area, forming vascular buds predominantly composed of immature type III collagen and proliferating fibroblasts in the matrix and repairing periodontal tissues at a rate of approximately 0.5 mm/day. After one week, the fractured collagen fibers reconnect, stabilizing the replanted tooth. By the second week, most of the principal fibers are repaired, and the periodontal ligament regains mechanical strength equivalent to approximately 2/3 of that of the original ligament.

Cell proliferation, migration, and differentiation are the foundations of tissue regeneration. Periodontal ligament cells constitute a heterogeneous population of cells, including osteoblasts, odontoblasts, cementoblasts, fibroblasts, and various precursor cells. Precursor cells possess multidirectional differentiation potential and, upon differentiating into osteoblasts, cementoblasts, and fibroblasts, participate in maintaining the stability of the periodontal microenvironment for soft and hard tissues. After tooth extraction, residual periodontal tissue remains at both the alveolar socket and the root surface, with more periodontal ligament stem cells (PDLSCs) on the alveolar socket side. Studies have shown that PDLSCs derived from the alveolar socket possess stronger differentiation and bone regeneration capabilities than those derived from the root surface, highlighting the importance of preserving residual periodontal ligament tissue within the alveolar socket in establishing new periodontal attachments during ITR.^20^

### Biomechanism of materials

The materials used for root-end filling and perforation repair during ITR directly contact periodontal tissues. The sealing ability, biocompatibility, and potential biological regulatory and antibacterial properties of these materials also influence the process of tissue repair and regeneration.^21^ Calcium silicate cements (CSCs), with formulations such as mineral trioxide aggregate (MTA), iRoot BP Plus, and Biodentine, are currently the most commonly used materials for root-end filling and perforation repair. CSCs exhibit excellent sealing properties and morphological stability and can release calcium, silicon and other inorganic ions in the fluid environment, affecting the biological behavior of surrounding cells.^22,23^ Research has shown that MTA can enhance the osteogenic differentiation potential of PDLSCs by activating the NF-κB and MAPK signaling pathways,^24^ while iRoot BP Plus can upregulate the autophagy level of bone marrow mesenchymal stem cells (BMSCs) and promote their osteogenic differentiation capacity.^25^ EMSs were performed in beagle models of apical periodontitis with MTA and iRoot BP Plus as root-end filling materials, and postoperative cone-beam computed tomography (CBCT) revealed that both materials achieved good apical sealing. However, iRoot BP Plus promoted better healing of the periapical bone and the formation of cementum-like, periodontal ligament-like, and bone tissue on the apical resection surface.^26^

Furthermore, during the healing process, CSCs release hydroxyl ions that permeate into the dentin, increasing the pH value of the surrounding environment and thereby exerting certain antibacterial effects against common pathogenic bacteria in infected root canals, such as *Enterococcus faecalis* and *Candida albicans*.^27,28^ It has been suggested that MTA exhibits certain antibacterial properties before complete healing, but after healing, the release of hydroxyl ions decreases, leading to the loss of its antibacterial properties.^29^ Notably, the antibacterial properties of other CSCs, such as iRoot BP and Biodentine, do not differ significantly from those of MTA.^30,31^ Currently, the assessment of the antibacterial properties of CSCs is limited to in vitro studies, with few reports on changes in pathogenic bacterial populations following in vivo application, thereby restricting their clinical significance.

## Case selection

### Indications

Teeth with pulpal and periapical diseases that remain unresponsive to nonsurgical RCT.

- Cases where the canal cannot be accessed for retreatment, such as teeth restored with crowns, post-and-core crowns, or bridges.
- Cases where the efficacy of nonsurgical treatment is uncertain, such as blocked canals and untreatable root canal perforations.^30,31^

Teeth unsuitable for EMS.

- Cases where establishing a surgical pathway for EMS is difficult, due to: those with restricted mouth opening, thick buccal fat pad, shallow vestibular sulcus, prominent external oblique ridge of the mandible, lingual inclination of the root apex, or posterior tooth positioning.^32,33^
- Cases where teeth are adjacent to vital anatomical structures, such as the maxillary sinus, inferior alveolar nerve canal, mental foramen, or lingual artery branches, in which EMS poses a risk of damaging, leading to postoperative complications such as paralysis or bleeding.^34,35^
- Cases where teeth have unrepairable root perforations, external root resorption, or cervical resorptive lesions, where apical surgery may cause excessive damage to alveolar bone and roots.^36,37^

Teeth with severe developmental abnormalities of anatomical structures, such as invaginated lingual grooves.^38,39^

*Patients strongly requesting retention of a single-rooted tooth with a vertical root fracture that does not extend through the entire root transection. In these cases, the tooth may be extracted and replanted after resin repair of the fracture*.^40,41^

### Contraindications

- Uncontrollable periodontal disease: Traditional guidelines consider periodontitis a contraindication for ITR^42^; however, current views suggest that root planning can be performed under direct vision during ITR to achieve better access and debridement than during conventional periodontal flap surgery. Some scholars believe that severe periodontitis is not an absolute contraindication for ITR.^43^ Comprehensive preoperative analysis of periodontal support is recommended to assess therapeutic efficacy.^44^
- Complete vertical root fracture.
- Inadequate supporting bone due to buccal or lingual alveolar bone defects, or insufficient alveolar bone in the furcation area, etc.
- Extensive loss of tooth structure in the affected tooth that cannot be restored functionally through restorative treatment.
- Difficulties in complete extraction due to a large root bifurcation angle or root deformities.
- Elderly patients with systemic complications and compromised tissue healing and regenerative capabilities.^13^

## Preoperative assessment

### General health assessment

Patients should be evaluated for cardiovascular disease, diabetes, and immune diseases; long-term use of oral or intravenous bisphosphonates; long-term oral or intravenous injection of high-dose corticosteroids; and the presence of artificial implants such as cardiac valves and artificial joints.

### Oral assessment

The condition of the prosthesis, root, periapical area, periodontal condition and adjacent structures should be assessed. CBCT examinations are recommended for assessing the number, distribution, and curvature of roots, especially in cases of root fractures. Preoperative RCT is generally recommended.

### Treatment plan

**Fig. 1:**
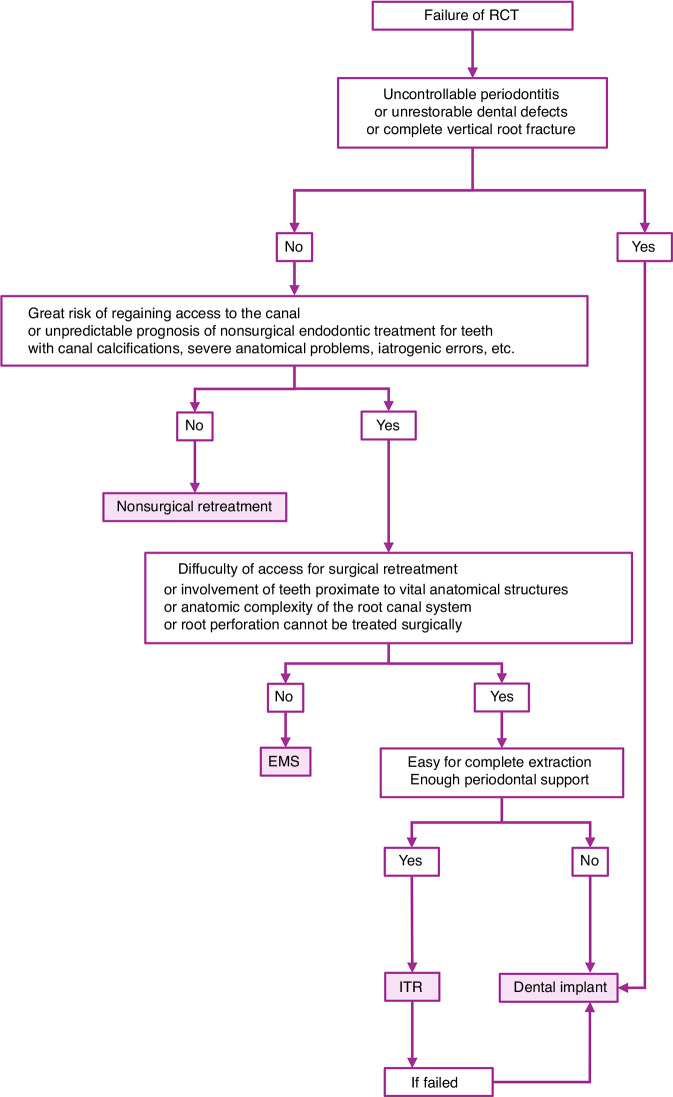
Decision-making flowchart for management of teeth with periapical diseases that fail to initial root canal treatment

For patients who have unsatisfactory results with nonsurgical retreatment or apical surgery, ITR is not the only option; extraction followed by implant restoration is also a viable option (Table 1 and Fig. 1).

**Table 1:** Advantages of ITR and dental implant

ITR	Dental implant
(1) Retention of natural tooth: Maintains proprioception, preserves gingival anatomy, and offers superior aesthetics. (2) Direct vision operation with a relatively controllable prognosis. (3) Lower treatment times and reduced economic costs. (4) Even after failed ITR, implant restoration remains a viable option.	(1) Mature technology with established standards for diagnosis and treatment. (2) Higher retention rate ( > 95%) for single dental implants compared to ITR (89%)^5^

## Clinical protocol

### Patient informed consent

Prior to any treatment, patients should be provided with complete information on their medical condition, treatment alternatives, cost, and prognosis. The routine informed consent includes a comprehensive overview of the procedures, risks and benefits of the treatment, as well as alternative treatment modalities. Once patients have been fully informed, the informed consent form should be signed before initiating treatment.

Generally, the affected teeth are pulpless which are brittle and susceptible to fracture during the process of extraction and inevitably cannot be retained. Thus, the operator must ensure the patient’s complete understanding of possible complications, including tooth fracture and loosening of crown restoration, based on complete communication prior to surgery.

### Management of surgical area

#### Preoperative periodontal maintenance

In preparation for surgery, comprehensive supragingival scaling and root planning are performed approximately one week prior to the scheduled procedure. Additionally, the administration of 0.12% chlorhexidine is recommended to minimize the risk of bacterial infection. A thorough evaluation of the gingival and periodontal condition of the affected teeth is conducted before surgical intervention.

#### Antibiotic prophylaxis

Antibiotic prophylaxis is not routinely recommended but is mandatory for medically compromised patients. The ESE guidelines recommend the use of prophylactic antibiotics for patients with a history of congenital heart disease, prosthetic heart valves, or infective endocarditis, as well as those who have received intravenous bisphosphonate therapy or undergone joint surgery within the past three months.^10^

#### Disinfection of the surgical area

The ESE guidelines recommend the use of an oral mucosa disinfectant, usually 0.12% or 0.2% chlorhexidine, for 1 minute.^10^

### Extraction of the affected tooth

**Fig. 2:**
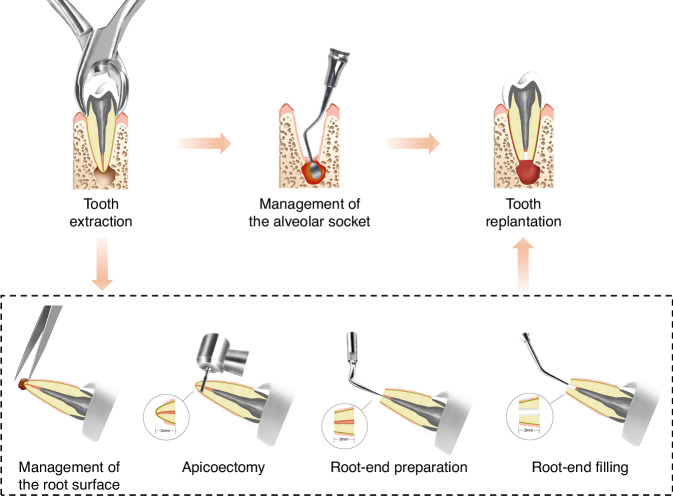
Schematic diagram of ITR

Tooth extraction is the most technically challenging aspect of the ITR procedure (Fig. 2). The tooth should be extracted intact with minimal damage to the surrounding periodontium. This critical step is essential for subsequent periodontium regeneration and successful implantation.

#### Instruments and techniques

Apical elevators, which can result in significant damage to the alveolar bone, periodontal ligament, and root surface cementum, are not recommended due to the greater risk of external root resorption and other postoperative complications. Instead, tooth extraction tools that cause less trauma to the periodontium, such as dental forceps, should be used.^45^ Preoperative CBCT is helpful for accurately assessing the shape and orientation of the roots. The appropriate type of extracting forceps should be selected based on tooth position and morphology. Placing the beak of the forceps coronal to the cementoenamel junction without contact with the cementum and alveolar fossa can prevent damage to the periodontal ligament. Approximately 2 mm of attached tissue above the alveolar crest is left, which allows the tooth forceps to slide downward.^46^ The tooth forceps should be used with careful and gentle pressure.^47^ A rubber band on the handle can aid in maintaining a constant pressure during the extraction process. It is not necessary to rush the extraction, and the alveolar socket can be slowly enlarged to the buccal-lingual side with a minor rotation before performing the dislocation. In cases where there are significant overfilling or separating instruments extend beyond the apical foramen, periapical radiographs should be taken after extraction to confirm that no gutta-percha or separating instruments remain in the alveolar fossa.

#### Advanced minimally invasive instruments and techniques

When a tooth exhibits cervical malabsorption and subgingival decay, traditional forceps may cause root fractures. Krug and colleagues investigated a minimally invasive tooth extraction system for anterior teeth and premolars, which involved placing a retainer drill in the root canal of the affected tooth, installing a retainer device on the adjacent tooth, and using a wheel shaft force to pull and dislocate the affected tooth. These procedures can avoid applying pressure on the periodontium of the affected tooth, and the incidence of postoperative complications is only 9.7%.^48,49^ However, the use of the minimally invasive tooth extraction system is currently limited to anterior teeth and premolars. Further research is needed to evaluate its effectiveness for molars.

Studies have demonstrated that prior disruption of the periodontal ligament connection can significantly reduce the difficulty of tooth extraction and improve the success rate. For instance, orthodontic extrusion can be performed 2–3 weeks before extraction for teeth with multiple roots and thick buccal cortical bone.^6^ During tooth extraction, Jang et al. first inserted a 15-gauge blade parallel to the periodontal ligament space along the direction of the root. By tapping the handle, the blade was wedged in the direction of the root tip to cut off the periodontal ligament and reduce resistance during tooth extraction. Additionally, it is suggested that an O-shaped rubber band be placed between the affected tooth and adjacent tooth 3–4 days before surgery to facilitate the release of the anchored connection between the affected tooth and the alveolar socket.^50^ However, retrospective analyses of the relationship between preoperative destruction of the periodontal ligament connection and postoperative healing of the periodontium are lacking. Meticulous planning is required before deciding on the optimal extraction method, including a comprehensive evaluation of the difficulty of tooth extraction and the periodontal status.

### Management of the alveolar socket

The key to effective alveolar socket management is maintaining the integrity of the periodontal ligament while properly removing diseased tissue. The extent and techniques of curettage in the alveolar socket depend on specific cases. Generally, apical granulation can be removed during tooth extraction, and residual minor granulomas can be tolerated especially when the lesion has been approximated to or invaded anatomic structures. Socket curettage can be performed under the magnification of a dental operating microscope with appropriate excavators or tissue forceps when large granulomas or cysts need to be removed. Scratching the alveolar socket with a curette is not recommended. During extraoral procedures, the protection of the alveolar socket with wet sterile gauze can help to prevent contamination with saliva or food residue.^10^

### Management of the extracted tooth

After complete extraction, removal of the granulation tissue on the tooth with tissue forceps is recommended. To minimize damage to critical periodontal cells on the root surface, it is advisable to refrain from direct scraping of the root surface. The root surface should be stained with methylene blue and carefully examined under high magnification with an operating microscope for the presence of anatomical structures such as fractures, perforations, and additional root canals or isthmuses. During the examination, dental tweezers or forceps should be held on the coronal side of the tooth to avoid damaging the periodontal ligament and cementum on the root surface.

The effect of extraoral management of teeth is dependent on the operation time and preservation medium, which are critical factors for determining the prognosis of replantation.^51^ The total extraoral operation time should be minimized and ideally controlled within 10-15 minutes to reduce the risk of periodontal ligament cell necrosis and root resorption after replantation.^10–55^ Pohl et al. identified extraoral operation time as a predictor of major complications, with the risk of postoperative complications increasing by approximately 1.7 times when the procedure time exceeded 15 minutes.^56,57^ The use of a liquid medium that preserves the activity of the periodontal ligament during the entire extraoral process is recommended. Hank’s balanced salt solution (HBSS) is the preferred preservative, with a pH close to 7 and osmolality of 270-290 mOsm/L, which provides suitable conditions for the survival of periodontal ligament cells.^58,59^ However, HBSS is relatively expensive and not widely available in some countries. Sterile normal saline (0.9% sodium chloride solution) and other electrolyte solutions, such as milk, are commonly used as preservation media, although they lack the trace elements and nutrients necessary for cell survival and can only be used as short-term storage media (approximately 2 hours).^60^ Systematic reviews have shown that HBSS, electrolyte solution, and milk can maintain more than 80% of periodontal ligament activity when used for up to 2 hours.^61^ During extraoral operations, the high-speed handpiece water outlet should be turned off to prevent contamination of the tooth. The crown of the tooth should be held with wet sterile gauze and dental forceps while continuously rinsing with the preservation medium to keep the root surface wet throughout the entire extraoral procedure. CSC root-end filling materials can be set and hardened in a humid environment, eliminating the need to dry the tooth during the root-end filling process.

### Management of the root apex

#### Apicoectomy

This procedure should be carried out with a magnification system. To resect the root end effectively, a Lindemann bur should be applied with a 45° angled handpiece, and piezosurgery is also suggested.^62^ Previous studies have shown that when the operating frequency of ultrasonic bone curettage coincides with the natural frequency of hard tissue, hard tissue is selectively resected without damaging soft tissue. The cavitation effect produced by ultrasonic bone curettage can reduce bleeding in the operation area while resulting in a smooth root-cutting surface that is conducive to the formation of new periodontal attachments.

Root resection 3 mm from the apex is generally recommended to reduce most of the apical ramifications and lateral canals. However, some cases require more than 3 mm of root-end resection, such as an apex in proximity to adjacent anatomical structures such as the mandibular canal and maxillary sinus, the presence of multiple accessory canals at the level of root resection, the presence and location of vertical microcracks, or excess lingual inclination of the root.^63^ Other factors, such as the level of crestal bone, the shape of the root, the thickness of the dentinal walls, and the apical extent of a post, may dictate the level of apical resection. The occurrence of apical root resorption may require less root resection. The apical extent of a post no more than 5 mm from the root apex may necessitate less coronal resection based on an evaluation of the accessory canals to ensure the depth of root-end preparation and root-end filling.

Furthermore, the root section should be perpendicular to the long axis of the tooth, with a bevel angle of less than 10°, to ensure optimal removal of anatomic variations, such as apical ramifications, accessory canals, and severe curves that are difficult to access during RCT. It is also important to completely remove the apical pathologic processes, reduce the exposure of dentinal tubules at the resected root surface, and preserve more buccal supporting bone.

#### Root-end preparation

During root-end preparation, an ultrasonic tip selected according to the tooth position is aligned parallel to the long axis of the root and along the root prominence to make a box-like cavity at least 3 mm deep with a specific retention form.^64^ During preparation, it is crucial to maintain gentle and intermittent movements while avoiding excessive pressure on the lateral walls to prevent microcracks. Additionally, it is important to maintain a dental wall thickness of approximately 2 mm. Notably, root canal depressions, isthmuses, or variant anatomies also require preparation to ensure complete sealing of the root canal system and periapical tissue. Spraying with a preservation medium such as HBSS and sterile saline is recommended during preparation to prevent tissue overheating. After preparation, any remaining gutta-percha should be compacted with a microcondenser.

#### Root-end filling

The root-end filling materials should be at least 3–4 mm thick. After the root-end filling material is compacted with a microplugger, excess material is removed with a wet cotton pellet to clean the resected root surface. The sealing properties, structural stability, and biocompatibility of root-end filling materials are crucial for the prognosis of ITR.^65^ MTA is regarded as the gold standard for root-end filling materials due to its excellent sealing ability and biocompatibility.^66^ However, some studies have shown that the success rate of MTA is limited in ITR teeth with C-shaped canals, which may be attributed to the difficulty in achieving hemostasis of the replanting area, the presence of anatomic variations such as the isthmus in the C-shaped canals, and the lengthy setting time of MTA.^50^ Blood fluids in the apical sealing area may affect MTA setting, mechanical properties, or sealing of microstructures such as the isthmus, which may lead to secondary infection. As a result, white MTA with a shorter setting time is recommended over gray MTA to avoid being washed out by the gingival sulcus. Furthermore, restricted mobility of the replanted tooth can reduce compression to the sealing material during occlusion to maintain a stable sealing effect.^63–67^ The development of new CSCs, such as iRoot BP Plus, has partially addressed the limitations of MTA, such as its long healing time and ability to handle difficulties, and it is currently widely used.^26–69^ Studies have demonstrated that iRoot BP Plus and MTA have no significant differences in terms of their biocompatibility or sealing ability.^70^ However, iRoot BP Plus exhibits superior marginal adaptability,^68^ and in animal models of periapical periodontitis and clinical trials, it has been shown to have a better healing effect than MTA.^71^ This result may be attributed to the anti-inflammatory properties of the nanoparticles in iRoot BP Plus, such as calcium silicate.

### Restoration and stabilization of the affected tooth

#### Tooth replantation and occlusion adjustment

When a blood clot in the alveolar socket hinders replantation, it can be gently removed with an aspirator, although direct contact with the alveolar socket should be avoided. The tooth should be reinserted into the alveolar socket with digital pressure along the long axis of the tooth. A gauze ball is placed on the occlusal surface if replantation is restricted, and the patient is asked to gently bite the tooth together to help the tooth settle in place.^72^ A 3 mm root-end resection generally leads to infraocclusion, so occlusal reduction is unnecessary. If there is premature contact with the affected tooth, whether the affected tooth is fully in place should be assessed first, and then the tooth should be reinserted if necessary. All occlusal interference should be removed.^10^ When the tooth is fully in place, the alveolar socket is compressed in a buccolingual direction to better fit the root. The patient is asked to gently bite on gauze or a cotton ball for five minutes to stabilize the affected tooth in cases with good periodontal health and interradicular bone intervals.

#### Stabilization technique for the affected tooth

The requirement and duration of stabilization depend on the mobility of the affected tooth after replantation. Currently, a periodontal splint is recommended for teeth with short roots or loss of alveolar bone when the degree of tooth mobility is II or III. However, this approach is not recommended for teeth with a degree of mobility of I. The ESE guidelines state that periodontal splints help promote healing of the periodontium of the affected tooth, and fine steel wires no greater than 0.3–0.4 mm in length should be bonded to the labial surface for stabilization.^10^ The duration of splinting varies from two weeks for most patients to up to 6 weeks for teeth with high mobility after surgical extrusion.

Some researchers have raised concerns that the use of periodontal splints with steel wires may affect physiologic tooth mobility and lead to ankylosis. In such cases, elastic fixation is recommended. This procedure involves a resin-bonded glass span seated in a prepared microgroove on the occlusal surface of the affected tooth and a dovetail on the adjacent tooth.^73^ This elastic splint can be maintained for more than a year without affecting normal physiological tooth mobility.

#### Additional management before replantation

Treating the root surface with specific drugs can improve periodontal reconstruction. Enamel matrix derivative (EMD) is a commercial product that contains 90% enamel matrix protein and promotes the reattachment of periodontal tissue, including the periodontal ligament, acellular cementum layer, and alveolar bone.^74–76^ Studies have shown that EMD can also decrease the survival of odontoclasts on the root surface and lower the risk of root resorption.^77^ The application of EMD significantly affects the success rate of intentional replantation.^65^ Radiographic examination revealed that root surfaces treated with tetracycline hydrochloride solution for five minutes and saline irrigation are free from root resorption.^78^ Some studies have found that preoperative treatment with minocycline hydrochloride promotes the regeneration of perforating fibers, which may be attributed to the antibacterial and anti-resorptive effects of tetracycline drugs on bone tissue.^78^ Overall, these laboratory and clinical studies verified that tetracyclines have antibacterial effects, promote alveolar bone formation and inhibit root resorption after replantation.

Some studies have demonstrated that pretreatment with enriched platelet products can also improve periodontal regeneration. Autologous platelet-rich plasma used for socket medicament of teeth affected with severe periodontitis can promote alveolar bone regeneration.^79^ The preoperative use of platelet-rich fibrin covered with a collagen membrane in a central incisor with severe periodontitis increased bone mass by approximately 87% at the one-year follow-up.^80^ Some reports have shown that platelet-rich fibrin can promote the regeneration of periodontal tissue and prevent postoperative ankylosis.^81^

### Treatment of special cases

#### Vertical root fractures

In ITR cases where vertical fractures are restricted in the root cross-section, sealing the fractures with resin cement extraorally is recommended to preserve the tooth.^82–84^ Moderate drying is performed to enhance the bonding effect of resin cement. Vertical root fractures involve the destruction of dentin, cementum, and the periodontal ligament. Resin can physically block communication between the pulp cavity and periodontium by sealing the fracture line without forming new cementum or periodontal ligament, which does not indicate true regeneration of dental and periodontal tissue. Fortunately, the development of bioactive materials such as CSC has improved the restoration of vertical root fractures. The microhardness and modulus of elasticity of Biodentine closely resemble those of natural dentin, making it an ideal fracture repair material. Furthermore, the initial setting time of Biodentine is only 15 minutes, and the final setting time is 45 minutes, resulting in a shorter extraoral operation period than that of MTA.^85^ Additionally, inorganic ions such as Ca/Si released from Biodentine create a beneficial environment for the attachment, proliferation, and differentiation of periodontal ligament stem cells, promoting biomineralization and the formation of new periodontal attachments.^86^ Many studies have shown the promising effect of ITR in treating vertical root fractures, but these studies are limited to individual case reports. A retrospective study revealed that the 12-month and 24-month survival rates of teeth with vertical root fractures after ITR were 83.3% and 36.3%, respectively.^87^

#### Cervical resorption lesions

Cervical resorption, which involves the loss of hard tooth tissue at the cementoenamel junction due to various factors, poses a challenge in avoiding resorption progression and achieving functional and aesthetically pleasing restoration. Intraoral conditions limit the field of vision and operating space, making it difficult to fully expose the lesion area. Periodontal flap surgery is often necessary but can cause further damage. ITR allows for direct visualization of the entire tooth, enabling treatment and a more definite prognosis.^48^ Treatment involves mechanical curettage and chemical debridement to remove granulation tissue and irregular parts of the absorption interface, exposing healthy dentin. The choice of repair material depends on the lesion location. For lesions located coronal to the cementoenamel junction, resin cement or glass ionomer is recommended, while CSC is recommended for apical lesions. Biodentine is a promising material for repairing dental tissue defects due to its mechanical properties that closely resemble those of natural dentin, faster setting, and superior biocompatibility and bioregulation, as mentioned above.^88^

## Postoperative management and follow-up

In the early stages following ITR, the periodontal tissue of the replanted tooth is in an open traumatic state, requiring consistent maintenance of oral hygiene. Apart from the replanted tooth and its adjacent teeth, routine cleaning should be performed on all other teeth. Typically, within the first week postoperatively, mouth rinses with 0.12% chlorhexidine solution should be used 2–3 times daily. A soft diet and avoiding chewing on the replanted tooth are recommended during the first week after surgery. Standard pain management protocols can be employed postoperatively, such as oral ibuprofen (300–600 mg/time, bid).^88^ The use of antibiotics postoperatively remains controversial. Some studies suggest that systemic use of antibiotics postoperatively is beneficial for preventing infection, with patients not receiving antibiotics having a 1.4-fold greater probability of developing complications such as root resorption.^89^ However, more studies and clinical guidelines advocate the cautious use of antibiotics.^10^ If an RCT is not performed prior to the ITR procedure, it must be carried out within 2 weeks postoperatively. Restoration and orthodontic treatments may commence after 6 months once the replanted tooth stabilizes.

### Duration and content of follow-up

Follow-up appointments are recommended at 2 weeks, 1 month, 3 months, 6 months, and 1 year postoperatively, with the option of extending follow-up intervals if necessary. During follow-up visits, any symptoms should be documented, and oral examination should include percussion, mobility, occlusion, palpation, periodontal probing, and assessment for sinus tract or fistula formation. Radiographic imaging of the periapical area is mandatory. Importantly, periodontal probing should not be performed prematurely after surgery to avoid disrupting the reconstruction of periodontal tissue.^90^

## Evaluation of outcomes

### Short-term outcomes (1 week after surgery)

- Improvement of preoperative clinical symptoms, including reduction or disappearance of pain, and healing of the gingival fistula.
- Assessment of periodontal tissue healing, including the ability to chew with the replanted tooth, pain or discomfort during chewing, clinical examination of gingival inflammation, tooth mobility, sensitivity to percussion, and tenderness.
- Detection of premature contacts between the replanted tooth and opposing teeth.

### Long-term outcomes (6 months after surgery and beyond)

- The absence of clinical symptoms, such as chewing pain and tooth mobility, and normal masticatory function.
- Clinical examination revealing the absence of gingival inflammation, significant recession, sensitivity to percussion, or tooth mobility.
- Periapical X-rays showing healing of the periapical lesions, absence of new lesions, and normalization of the periodontal ligament space.
- No signs of root resorption or ankylosis.

## Prognosis and complications

The types of periodontal tissue healing following ITR include periodontal ligament healing, surface resorption healing, inflammatory resorption, and replacement resorption.^63^ (1) Periodontal ligament healing is the best outcome that frequently occurs after immediate replantation and involves either reattachments or new attachments, both of which rely on the presence of surviving periodontal ligament cells on the root surface and within the alveolar socket.^38–51,55–93^ (2) Surface resorption healing occurs when there is limited damage to the root surface and periodontal ligament, and healing occurs after repair by adjacent intact periodontal ligaments. Pure surface resorption is self-limiting and can be repaired by the formation of new cementum and periodontal ligaments, resulting in new reattachment. (3) Inflammatory resorption occurs when inflammation persists after tooth replantation, leading to significant resorption of alveolar bone and progressive root resorption. The rapid progression of inflammatory resorption can result in the failure of ITR. A meta-analysis revealed that among 838 replanted teeth, the overall success rate was 88%, with 11% failing due to root resorption, which was the primary cause of replantation failure.^94^ (4) Tooth ankylosis and replacement resorption occur when severe damage to the root surface or periodontal ligament leads to direct contact between the root and alveolar bone, resulting in fusion and bone repair and characterized clinically by the absence of tooth mobility and radiographically by the disappearance of the periodontal ligament space.

Prolonged extraoral time, root desiccation, and reduced vitality of periodontal ligament cells can impact tissue healing, leading to complications such as external root resorption, root fusion, and postoperative infection.^95^ Additionally, factors such as the extent of periapical lesions, periodontal condition, patient age, level of expertise in extraction technique, alveolar socket preparation, and choice of root-end filling materials can affect the prognosis of replantation.^43,45–51,55–65^ Recent systematic reviews and meta-analyses indicate an overall average survival rate of approximately 88% for ITR,^96^ with the success rate varying over time. The short-term success rate (< 6 months) is approximately 90%, decreasing to 65% to 80% at 12 to 36 months and stabilizing beyond 36 months of follow-up.^57^ Asymptomatic replanted teeth may continue to be observed, and their impact on subsequent alternative implant treatment planning should be considered.

## Conclusion and expectations

As a last resort for preserving teeth with periapical lesions, ITR provides a viable option for maximizing the retention of natural teeth. The affected teeth are generally brittle and are susceptible to fracture during the process of extraction, therefore, endodontists should be well-versed in the indications and clinical management of this technique to enhance its long-term success.

It is worth noting that current research on ITR mainly relies on case reports and retrospective studies, and there is a lack of high-level evidence such as randomized controlled trials. In order to obtain more convincing conclusions, further studies with larger sample sizes and longer observation periods are needed. Meanwhile, it is necessary to explore the key factors that affect the treatment outcomes and develop artificial intelligence evaluation systems, so as to improve the success rate and predictability of ITR.
